# Pre- and post-diagnosis physical activity is associated with survival benefits of colorectal cancer patients: a systematic review and meta-analysis

**DOI:** 10.18632/oncotarget.10603

**Published:** 2016-07-18

**Authors:** Wenrui Wu, Feifei Guo, Jianzhong Ye, Yating Li, Ding Shi, Daiqiong Fang, Jing Guo, Lanjuan Li

**Affiliations:** ^1^ State Key Laboratory for Diagnosis and Treatment of Infectious Diseases, The First Affiliated Hospital, School of Medicine, Zhejiang University, Hangzhou, China; ^2^ Collaborative Innovation Center for Diagnosis and Treatment of Infectious Diseases, Hangzhou, China

**Keywords:** physical activity, pre-diagnosis, post-diagnosis, colorectal cancer

## Abstract

**Objective:**

Physical activity is associated with reduced risk of colorectal cancer. However, whether physical activity could impart cancer patients’ survival benefits remains uncertain. The aim of this study is to systematically evaluate the relationship between physical activity and colorectal cancer mortality.

**Results:**

Our meta-analysis included 11 studies involving 17,295 patients with a follow-up period ranging from 3.8 to 11.9 years. Results indicated that physical activity was inversely associated with overall (RR = 0.81, 95% CI = 0.72–0.91) and colorectal cancer-specific mortality (RR = 0.79, 95% CI = 0.71–0.89) before the diagnosis of cancer, respectively. For physical activity after diagnosis, the pooled RRs of colorectal cancer-specific and total mortality were 0.77 (95% CI, 0.63–0.94) and 0.71 (95% CI, 0.63–0.81), respectively. Similar inverse associations between exercise and prognosis were found among colorectal cancer survivors who had high-level exercise compared with those who had low-level exercise or were inactive. There was no obvious evidence for publication bias among studies.

**Materials and Methods:**

We performed a systematic data search in PubMed, Cochrane Library databases and Web of Science for relevant articles before Jan 2016. We adopted adjusted estimates to calculate pooled relative risks (RRs) with 95% confidence intervals (CI) by the random-effects model. The publication bias was assessed by Begg's test.

**Conclusions:**

Our meta-analysis provides comprehensive evidence that physical activity, whether before or after the diagnosis of colorectal cancer, is related to reduced overall and cancer-specific mortality. Our findings may have significant public health implications and more prospective randomized clinical trials should be warranted to certify this protective association.

## INTRODUCTION

Colorectal cancer (CRC) still represents one of most common malignances and a leading cause of cancer-related deaths globally, which accounts for over 1.2 million new colorectal cancer patients and 608,700 deaths in 2008 [1]. Due to advances in early diagnosis and comprehensive treatments, the 1-year relative survival rates of CRC have reached 83.4% [2]. However, 5-year survival rates were still 64.9% and long-term prognosis of CRC was poor [3]. Therefore, there is an urgent need to adopt measures to reduce the incidence of cancer recurrence and mortality among colorectal cancer survivors.

Accumulating evidence from epidemiologic studies has indicated that some modifiable lifestyle factors, such as physical activity, were linked with reduced incidence of colorectal cancer and a higher level of exercise among cancer survivors might contribute to lots of health benefits for these individuals [4–6]. However, the relationship between physical activity and prognosis of colorectal cancer remains inadequately understood. Although several prospective cohort studies had found the inverse association between exercise and total or cancer-specific mortality among patients [7–11]. Controversies still remained regarding different timing point of physical activity assessment and survival outcome measurements. The results from studies that post-diagnostic exercise was related to favorable survival outcomes were consistent [8, 10, 12, 13]. For example, Kuiper et al. [13]. reported that post-diagnosis physical activity could reduce cancer–specific and overall mortality among CRC patients. However, whether the pre-diagnostic physical activity could affect CRC survival was less conclusive. Some studies reported a favorable relationship between pre-diagnostic activity and prognosis of CRC [8, 14], while others indicated no significant association. For example, Meyerhardt et al. [10] reported that physical activity before the diagnosis was not associated with better prognosis among CRC survivors. Furthermore, it remains uncertain whether the beneficial effect of physical activity on CRC survival was influenced by other factors.

Considering these controversial contexts, we performed a systematic review and meta-analysis based on existing evidence to test and determine whether pre- or post- physical activity could influence cancer-specific and total mortality in order to better understand the effects of physical activity interventions on prognosis of CRC survivors.

## RESULTS

### Literature search and study characteristics

**Figure 1:**
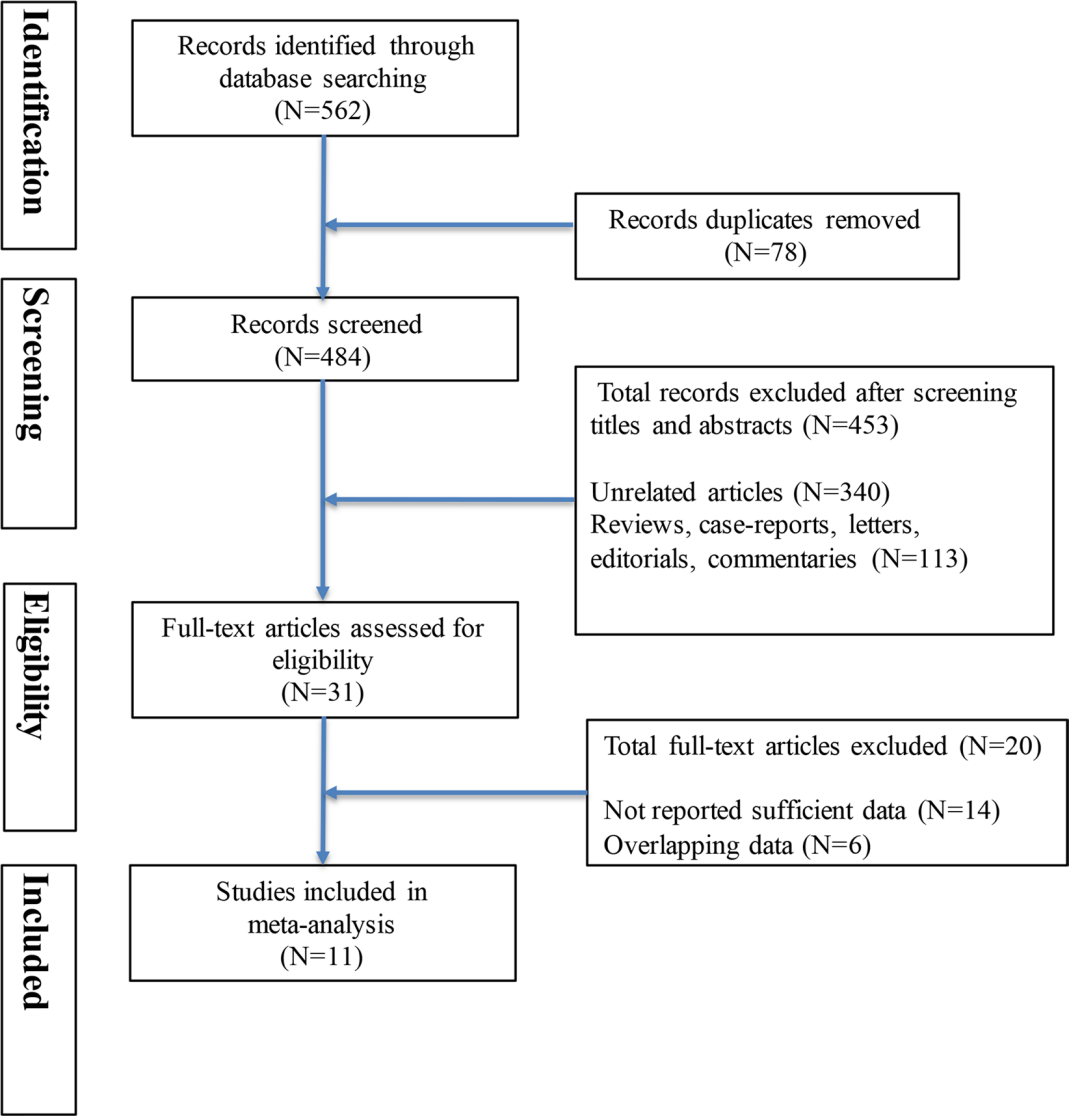
Flow diagram summarizing study identification and selection

Our literature search yielded 562 articles in PubMed, Cochrane Library databases, Web of Science and manual search. After screening titles and abstracts of articles, 31 articles were reviewed with full texts. Finally, 11 prospective articles [7–17] were included in this meta-analysis and other twenty articles were excluded because they provided insufficient or overlapping information. Details of the selection process for studies were shown in Figure 1. The total number of participants in the meta-analysis was 17,295 with a follow-up period ranging from 3.8 to 11.9 years (Table 1). Among them, eight articles reported the association between physical activity before the diagnosis and CRC outcomes and seven studies presented CRC mortality in relation to physical activity after the diagnosis. Exposure assessment of physical activity was based on self-reported or interview-based across studies. The level of physical activity was accessed as MET-hours per week or hours per week. The overall methodological quality of included studies was moderate to high using the Newcastle–Ottawa scale quality tool (Table 2).

**Table 1:** Characteristics of included studies assessing the relationship between physical activity and mortality of colorectal cancer patients

Study	Design	Location	Total subjects	Follow-up period (years)	CRC Stage	Timing assessment of physical activity	Confounding variables adjusted
Romaguera et al. 2015	Cohort	Europe	3292	4.2	I, II, III, IV, unknown	Pre-diagnosis	1, 3, 4, 5, 6, 7, 14
Hardikar et al. 2015	Cohort	USA	1309	6.1	0/I, II, III	Pre-diagnosis	1, 2, 3, 6, 8, 7, 14
Arem et al. 2015	Cohort	USA	3,797	7.8	localize, regional unknown	Pre-diagnosis, Post—diagnosis	1, 3, 4, 5, 7, 8, 9, 10, 11, 12, 13
Boyle et al. 2013	Cohort	Australia	879	5.6	I, II, III, IV, unknown	Pre-diagnosis	1, 2, 3, 7, 8, 15, 16
Campbell et al. 2013	Cohort	USA	2,262	6.8	localized regional	Pre-diagnosis, Post—diagnosis	1, 2, 3, 6, 7, 8, 13, 14, 17
Kuiper et al. 2012	Cohort	USA	1339	11.9	localized regional	Pre-diagnosis, Post—diagnosis	2, 3, 6, 7, 8, 19, 20, 21, 22
Baade et al. 2011	Cohort	Australia	1825	4.9	I, II, III, unknown	Post—diagnosis	1, 2, 3, 5, 6, 7, 8, 9, 10, 11
Meyerhardt et al. 2009	Cohort	USA	661	8.6	I, II, III, Missing (not metastatic)	Post—diagnosis	2, 3, 4, 5, 7, 8, 14, 18, 24
Meyerhardt et al. 2006(NHS)	Cohort	USA	573	9.6	I, II, III	Pre-diagnosis, Post—diagnosis	2, 3,4, 5, 7, 8, 11, 14, 18, 24
Haydon et al. 2006	Cohort	Australia	526	5.5	I, II, III, IV, unknown	Pre-diagnosis	1, 2, 3
Meyerhardt et al. 2006(CALGB)	Cohort	USA	832	3.8	III	Post—diagnosis	1, 2, 3, 4, 8, 23, 24, 25, 26

Abbreviations: 1. sex, 2. age, 3. tumor stage, 4. tumor grade, 5. tumor site, 6. educational, 7. smoking, 8. BMI, 9. surgery, 10. radiation, 11. chemotherapy, 12. self-reported health, 13. time of watching TV/sitting, 14. year of diagnosis, 15. socioeconomic status,16. diabetes, 17.red meat intake, 18. change in body mass index before and after diagnosis, 19. study arm, 20. alcohol, 21. hormone therapy use, 22. ethnicity, 23. presence of clinical perforation or obstruction at time of surgery, 24. time from diagnosis to physical activity measurement, 25. baseline performance status, 26. baseline CEA.

**Table 2:** Newcastle-Ottawa scale for assessment of quality of in included Cohort studies

Author	Quality assessment criteria								Overall Quality Score (max = 9)
	selection			Comparability			Outcome		
	Representativeness of exposed cohort?	Selection of the non-exposed cohort?	Ascertainment of exposure?	outcome of interest was not present at start of study?	Study control for age/gender and additional factor?	Assessment of outcome?	Was follow-up long enough for outcome to occur?	Adequacy of follow-up of cohorts?	
Romaguera et al. 2015	*	*	*	*	**	*	*	*	9
Hardikar et al. 2015	*	*	*	*	**	*	*	−	8
Arem et al. 2014	*	*	*	*	**	*	*	−	8
Boyle et al. 2013	*	*	*	*	**	*	*	*	9
Campbell et al. 2013	*	*	*	*	**	*	*	−	8
Kuiper et al. 2012	−	*	*	*	**	*	*	−	7
Baade et al. 2011	*	*	*	*	**	*	−	−	7
Meyerhardt et al. 2009	−	*	*	*	**	*	*	*	8
Meyerhardt et al. 2006 (NHS)	−	*	*	*	**	*	*	−	7
Meyerhardt et al. 2006 (CALGB)	−	*	*	*	**	*	*	*	8
Haydon et al. 2006	*	*	*	*	**	*	*	*	9

Each asterisk represents if individual criterion within the subsection were fulfilled.

### Association of physical activity with colorectal cancer mortality

**Figure 2:**
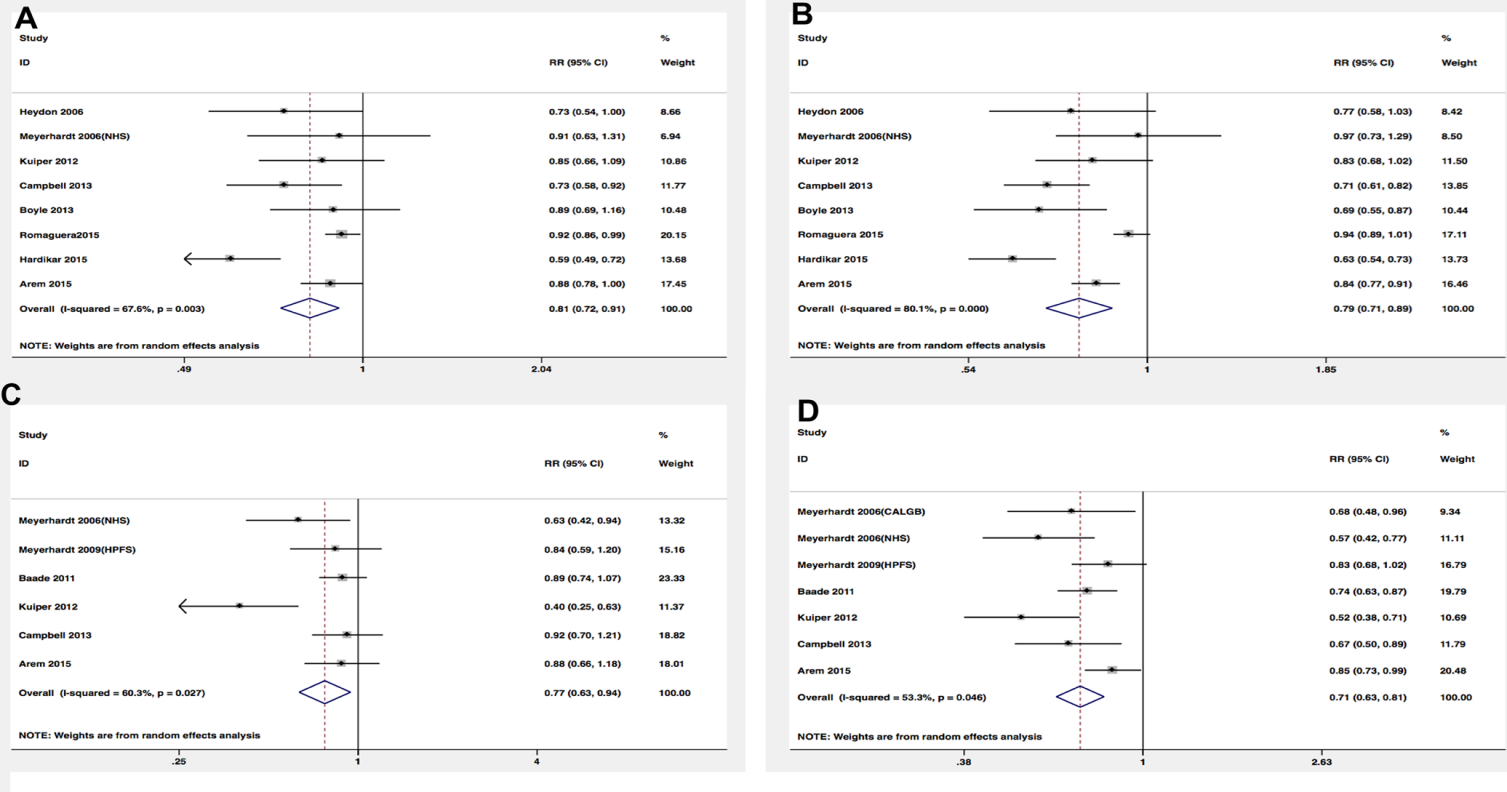
Relative risks for the association between physical activity and survival among exercise patients VS non-exercise patients (**A**) Pre-diagnosis and CRC-specific mortality; (**B**) Pre-diagnosis and overall mortality; (**C**) Post-diagnosis and CRC-specific mortality; (**D**) Post-diagnosis and overall mortality.

**Figure 3:**
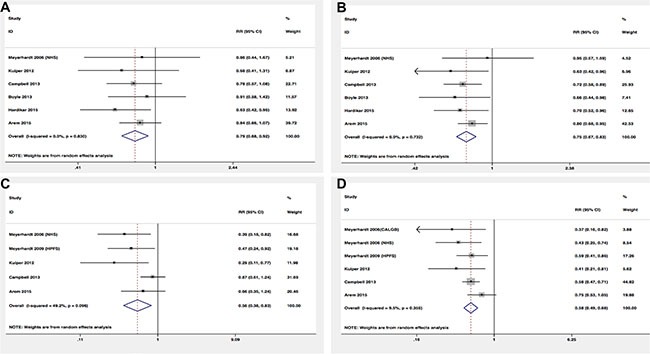
Relative risks for the association between physical activity and survival among high-level VS low-level (**A**) Pre-diagnosis and CRC-specific mortality; (**B**) Pre-diagnosis and overall mortality; (**C**) Post-diagnosis and CRC-specific mortality; (**D**) Post-diagnosis and overall mortality.

Among eight studies that assessed physical activity before the diagnosis in relation to prognosis, we observed that exercisers had lower colorectal cancer-specific mortality and overall mortality (RR = 0.81, 95% CI 0.72–0.91; RR = 0.79, 95% CI 0.71–0.89, respectively) (Figure 2A, 2B). However, there was some evidence of significant heterogeneity among the studies for specific (*P* = 0.003, *I*^2^ = 67.6%) and overall mortality (*P* = 0.000, *I*^2^ = 80.1%). In order to test the robustness of our results, we also performed sensitivity analysis and the associations were not materially altered by an omission of one study at each time. Similar inverse associations were also observed for different levels of exercise. Six studies presented results on high vs low levels of pre-diagnosis physical activity and mortality. The pooled RR of 0.79 (95% CI, 0.68–0.92) and 0.75 (95% CI, 0.67–0.83) were found for cancer-specific and overall mortality, respectively (Figure 3A, 3B). There was no evidence of existing heterogeneity across studies. We found no obvious evidence of publication bias in any analyses by Begg's test.

In terms of physical activity after diagnosis, seven studies presented the relationship between prognosis of cancer survivors and physical activity levels after the diagnosis. Similar protective effects were also observed among analyses. Post-diagnosis physical activity was associated with more favorable CRC-specific survival (RR = 0.77; 95% CI, 0.63–0.94) (Figure 2C) and better overall survival (RR = 0.71; 95% CI, 0.63–0.81) (Figure 2D) compared with non-exercisers. However, some evidence of heterogeneity among studies was observed. Therefore, we performed sensitivity analysis to test whether our results were robust. Removing anyone study at each time did not affect the overall outcomes substantially. When comparing high versus low levels physical activity after the diagnosis, we found the higher level of post-diagnosis physical activity was associated with lower CRC-specific mortality (RR = 0.56; 95% CI, 0.38–0.83) (Figure 3C) and overall mortality (RR, 0.58; 95% CI, 0.49–0.68) (Figure 3D). No evidence of heterogeneity was found among studies.

## DISCUSSION

Based on 11 prospective cohort studies, our comprehensive meta-analysis indicated that both pre-diagnosis and post-diagnosis exercises were related to reduced risk of all-cause and cancer-specific death among cancer survivors. Furthermore, the higher levels of exercise also exerted survival benefits among CRC patients compared with low levels. Despite significant heterogeneity was existed among studies assessing patients between exercise and no-exercise, sensitivity analyses demonstrated the robustness of our overall outcomes. Our meta-analysis supported the hypothesis that exercise could reduce cancer mortality and improve prognosis, which was consistent with previous studies [18]. Given the paucity of data, whether changing in physical activity from before to after diagnosis was associated with favorable survival was still unclear, which might be more clinically relevant for patients and doctors. One study performed exploratory analysis, which consisted of 573 colorectal cancer women with stage I to III [10]. Compared to women with no change of their activity, those who increased their activity levels had better cancer-specific and overall prognosis, independent of the exercise level before diagnosis (HR = 0.48, 95% CI, 0.24–0.97; HR = 0.51,95% CI, 0.30–0.85, respectively) [10]. This indicated that patients could still benefit from exercise, even for those were in physical inactivity before diagnosis. Additional results from RCTs are needed to verify this association ultimately. In order to better characterize and support the biologic relationship between exercise and cancer survival, several observational studies recently focused on whether different molecular features of colorectal cancer could modify the associations between physical activity and survival [14, 19, 20]. Among 382 patients with PTGS2-positive colorectal cancer, the highest level of physical activity was associated with an 82% lower cancer–specific mortality compared with the least level. However, this protective association was disappeared among 223 patients with PTGS2-negative tumors [19]. Similarly, in another study, nuclear CTNNB1 negative colorectal cancer individuals who were active in exercises had a 67% lower risk of cancer–specific death compared with less active patients after the diagnosis, whereas post-diagnosis exercise was not related to lower mortality among patients who were nuclear CTNNB1-positive [20].

In spite of the protective link between physical activity and the incidence of colorectal cancer [18, 21], underlying biologic mechanisms among the inverse relationship are less apparent. Several potential plausible mechanisms are posited to explain this protective influence of physical activity on prognosis of cancer survivors. For example, Some intervention studies reported exercise may reduce plasma insulin levels and increase insulin sensitivity [22, 23], thus attenuating hyperinsulinemia [24]. It was well documented that the high levels of insulin and insulin-like growth factors (IGF) were associated with malignancy growth, angiogenesis, and metastasis *in vitro* and animal studies [25–27]. Reduction of insulin and IGF levels could reduce the risk of CRC and improve survival outcomes [28, 29]. In addition, physical activity may reduce systemic levels of inflammation and improve immune function, which was supported by recent studies [29–31]. It was reported that regular exercise reduces the volumes and recurrence of five different tumors in mice through nature killer cell activation [30]. Furthermore, physical activity also improved tolerance of surgery or adjuvant treatment and reduced risks of comorbid conditions [8].

The strength of our systematic analysis was included comprehensive prospective studies and large numbers of patients, together with the assessment of the difference among survival benefits between pre- and post-diagnosis. However, our meta-analysis had several limitations that merit further consideration. First, our meta-analysis pooled data from observational studies, which had some methodical shortcomings and were prone to cause reverse causality. CRC patients who had less physical activity may be due to the severity of the disease at the time of physical activity assessment. In order to minimize this possibility of reverse causation, we adopted adjusted RRs and excluded patients at CRC IV stage. Second, exposure assessment of physical activity was self-reported or interview-based, which inevitably introduced some measurement errors into studies. Accurate measurement of physical activity is needed in further epidemiologic studies. Third, there was a significant heterogeneity in our analysis. Although we performed sensitivity analyses and overall outcomes did not materially alter, results of our analyses need to be interpreted with caution.

This meta-analysis strengthens the protective role of physical activity as a promising candidate for CRC therapeutics among cancer survivors. However, it remains unknown that how effects of physical activity, including duration, intensity and types of activity, will vary on survival outcomes and which type of CRC among patients is most likely to benefit from exercises. In addition, it is largely unclear whether the effect of post-diagnostic physical activity on CRC survival is influenced by patients’ pre-diagnostic levels. In future, large prospective observational studies and RCTs with accurate assessment of physical activity and sufficiently long follow-up durations are needed to elucidate those questions.

In conclusion, the current systematic analysis showed that physical activity, both before and after colorectal cancer diagnosis, was associated with a lower risk of mortality and improved survival of CRC survivors. This finding extended our current evidence and supported the promising role of physical activity on better prognosis of cancer survivors. In the meantime, it may be rational to recommend cancer survivors to adopt moderate levels of physical activity in their lifestyle. Further researches, however, are warranted to examine this beneficial effect before a definite conclusion can be reached.

## MATERIALS AND METHODS

This meta-analysis was reported according to the Preferred Reporting Items for Systematic Reviews and Meta-Analysis (PRISMA) guidelines [32].

### Search strategy

We (WWR and GFF.) independently performed a literature search in PubMed, Cochrane Library databases and Web of Science for all relevant studies before Jan 2016. The following keywords were adopted in this search procedure: ‘colorectal cancer’, ‘colon cancer’, ‘rectal cancer’, ‘colorectal adenocarcinoma’, ‘exercise’, ‘physical activity’, ‘motor activity’, ‘survival’, ‘prognosis’, ‘recurrence’, and ‘mortality’. Two authors screened the titles and abstracts of articles identified in the search and excluded unrelated articles independently. We further scrutinized the remaining full articles and lists of references in lest we ignored some additional relevant studies.

### Eligibility criteria

Two authors performed eligibility assessment in a standardized manner independently. The inclusion criteria were set in advance. The article was included in this meta-analysis if they met the following criteria:(1) original article that evaluated association between physical activity and CRC; (2) study that reported relative risk (RR) with 95% CI; (3) the exposure of physical activity was assessed before or after diagnosis of CRC; (4) the interesting outcomes were overall mortality or colorectal cancer-specific mortality. If there were multiple publications which were overlapped or duplicated, we extracted the information from the most comprehensive study. Any discrepancies in the study selection were resolved by discussions or reviewing of the original article.

### Data extraction and quality assessment

Two authors (WWR and GFF.) extracted data from included articles independently and the results were crosschecked. Following information was extracted in each individual article: authors, years, design, location, the number of total subjects, follow-up period, colorectal cancer stage, timing assessment of physical activity, and confounding variables adjusted. We contacted authors for important information by email if the required data were unavailable.

The Newcastle-Ottawa Scale was applied for quality assessment in order to understand the risk of bias among articles. Two authors independently performed all methodological quality of eligible studies. Any disagreements were resolved by discussions or with a third author.

### Statistical analysis

We calculated pooled RRs for physical activity before and after diagnosis in relation to overall or CRC-specific mortality using the DerSimonian and Laird random-effects models [33], since the heterogeneity across studies was considerable. We adopted adjusted RRs reported in studies for meta-analysis in order to reduce the bias. Two methods were applied to calculated heterogeneity among each study [34]. Cochran's Q statistic and *I*^2^. If *P* value < 0.1 or *I*^2^ values > 50%, statistically significance for heterogeneity was considered [34, 35]. The sensitivity analysis was performed through eliminating each study every time and calculated the pooled RRs for remaining studies in order to evaluate whether the results were affected by a single study. Potential publication bias was assessed by Begg's test. In addition, we investigated whether the degree of physical activity would influence survival outcomes of CRC patients. A *P* level < 0.05 (except for Cochran's *Q* test) was considered statistically significant and all *P* values were two tailed. All statistical analyses were conducted using Stata software version 13.0 (Stata Corporation, College Station, Tex).
